# Successful Treatment of Hamstring Tendinopathy in a Nonathlete with Ultrasound-Guided Injection to the Ischial Tuberosity: A Case Report

**DOI:** 10.3390/reports9030204

**Published:** 2026-06-26

**Authors:** Kunitaro Watanabe, Chihiro Akizawa, Ryuji Sawada, Mieko Chinzei, Kiyoshi Moriyama

**Affiliations:** Department of Anesthesiology, Kyorin University School of Medicine, Tokyo 181-0004, Japan; chrchrchr1229@gmail.com (C.A.); waterbouzu05@gmail.com (R.S.); chinzeimieko@gmail.com (M.C.); mokiyo@ks.kyorin-u.ac.jp (K.M.)

**Keywords:** buttock pain, sciatica, piriformis syndrome, lumbar disc herniation, chronic pain

## Abstract

**Background and Clinical Significance**: Proximal hamstring tendinopathy can occur not only in athletes but also in nonathletes when daily activities impose repetitive tensile or compressive loading at the ischial tuberosity. Because symptoms often resemble piriformis syndrome or lumbar pathology, diagnosis may be delayed; **Case Presentation**: A woman in her twenties developed buttock pain during desk work. Lumbar MRI was normal, and piriformis blocks provided only temporary relief. Localized tenderness at the ischial tuberosity, pain provocation during sitting, and positive provocation tests suggested proximal hamstring tendinopathy. Ultrasound showed a mildly hypoechoic area at the tendon insertion without definite thickening or tear. Ultrasound-guided injection of levobupivacaine and dexamethasone produced immediate but temporary relief. She continued receiving injections every two weeks, combined with stretching, hip-lift strengthening, and reduced sitting. After 18 injections, her pain improved from a numerical rating scale score of 10 to 0–1; **Conclusions**: This case demonstrates that proximal hamstring tendinopathy can develop in nonathletes due to lifestyle-related mechanical loading. Characteristic clinical findings and ultrasound evaluation are essential for diagnosis, and ultrasound-guided injection combined with exercise and activity modification provided sustained symptom improvement.

## 1. Introduction and Clinical Significance

Proximal hamstring tendinopathy (PHT) is a chronic pain condition involving tendon insertions of the semimembranosus, semitendinosus, and biceps femoris muscles at the ischial tuberosity. It is commonly recognized as a sports-related overuse injury, particularly among athletes engaged in repetitive hip flexion and extension activities such as long-distance running, sprinting, and cycling [1]. However, recent reports indicate that PHT can also occur in nonathletes owing to compressive and tensile loading of the tendon from sustained anterior pelvic tilt, prolonged sitting, or walking in high heels [1].

The ischial tuberosity serves as the common origin of the proximal hamstring tendons and is subjected to substantial mechanical stress during hip flexion under load. Repetitive tensile and compressive forces at this enthesis can lead to collagen disorganization, tendon thickening, and peritendinous edema, which are characteristic pathological features of PHT. In particular, the semimembranosus tendon is frequently involved because of its broad footprint and its greater exposure to compressive loading against the ischial tuberosity during deep hip flexion. These biomechanical factors help explain why PHT may also develop in individuals without athletic backgrounds [2].

Clinically, PHT presents as deep buttock and posterior thigh pain, requiring differentiation from lumbar disc herniation, piriformis syndrome, and sciatica. Because imaging findings may be subtle, a diagnosis requires a comprehensive assessment, including history taking, physical examination, and ultrasonographic evaluation [1].

We present a case of PHT in a young woman without an athletic background who was successfully treated with ultrasound-guided local injection to the ischial tuberosity.

## 2. Case Presentation

A woman in her twenties developed bilateral buttock pain during desk work and consulted orthopedic and neurology clinics. Lumbar Magnetic resonance imaging (MRI) revealed no abnormalities, and she was referred to our pain clinic with a diagnosis of piriformis syndrome. She reported pain extending from the buttocks to the posterior thighs, which worsened during hip flexion. Two piriformis muscle blocks provided only temporary relief.

**Figure 1:**
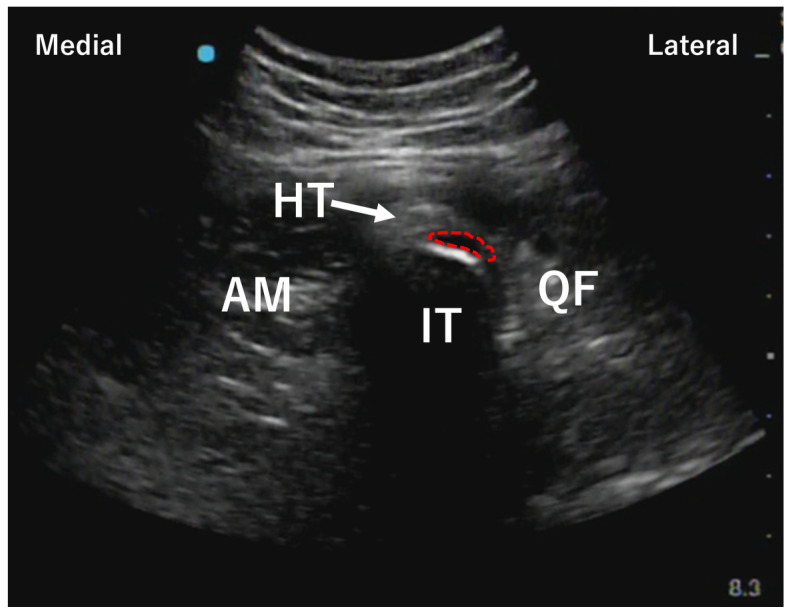
Ultrasound image of the ischial tuberosity showing the proximal attachment of the hamstring tendon. A short-axis ultrasound image at the ischial tuberosity demonstrated hypoechoic thickening of the proximal semimembranosus tendon. The probe was positioned in a transverse (short-axis) orientation, allowing visualization of the tendon cross-section and adjacent structures. A mildly hypoechoic (Red dot line) area is visible at the enthesis, without evidence of tendon thickening or tear. IT: ischial tuberosity; HT: hamstring tendon; AM: adductor magnus; QF: quadratus femoris.

The patient’s buttock pain recurred after prolonged sitting in the waiting room, and localized tenderness over the ischial tuberosity raised suspicion of hamstring insertional tendinopathy. Although she had no athletic background, her previous job in a large shopping mall required walking more than 20,000 steps per day in high-heeled shoes. After the onset of buttock pain, she transitioned to a desk-based job; however, prolonged sitting again provoked similar symptoms. Both the Puranen–Orava test and the bent-knee stretch test were positive, supporting the diagnosis of proximal hamstring tendinopathy. A pelvic or ischial tuberosity MRI was not obtained because the patient declined additional imaging. A mildly hypoechoic area was observed at the hamstring tendon insertion on the ischial tuberosity, but no definite tendon thickening or tear was identified (Figure 1).

Ultrasound-guided injection of 0.2% levobupivacaine (4 mL) and 3.3 mg dexamethasone into the ischial tuberosity resulted in immediate but temporary pain relief. As illustrated in Figure 1, the procedure was performed in a short-axis orientation using a cross-needle approach, allowing accurate advancement of the needle into the proximal hamstring tendon. The patient was instructed to perform pain-free stretching and hip-lift strengthening exercises and to avoid prolonged sitting.

Pain intensity was recorded at each visit. At the first injection, the patient reported a baseline Numerical Rating Scale (NRS) of 10, which decreased to NRS 0 immediately after the procedure. Dexamethasone was administered at both the first and second injections. At the second visit, pain intensity had decreased to NRS 5. At the third visit, the NRS was 7, and dexamethasone was again administered. From the fourth injection onward, dexamethasone was used approximately once per month, while levobupivacaine alone was injected at the remaining visits. During the early phase of treatment, NRS values ranged from 5 to 7, followed by gradual improvement to NRS 3–4 in the mid-phase. In the later phase, pain intensity further improved to NRS 0–1, with minimal interference in daily activities. A total of 18 ultrasound-guided injections were performed over the treatment course, after which symptoms had stabilized at NRS 0–1.

Block injections were continued approximately every two weeks, and her symptoms stabilized without recurrence after eight months.

## 3. Discussion

Diagnosing PHT can be challenging, particularly in nonathletes, in whom clinical recognition is often delayed. In this case, MRI revealed no abnormalities, such as disc pathology or piriformis hypertrophy, and piriformis blocks provided limited benefit. The presence of ischial tuberosity tenderness, symptom recurrence during sitting, and pain exacerbation with hip flexion strongly suggested PHT.

Goom et al. reported that PHT occurs not only in athletic populations but also in individuals who do not participate in sports, and that bilateral symptoms are not uncommon in this non-athletic demographic [3]. Although PHT is often described in athletic populations, clinical commentaries have emphasized that it also occurs in individuals who do not participate in sports, and bilateral symptoms are not uncommon in this group. In some non-athletic patients, compressive load simply from sitting has been identified as the primary load-inciting factor, which aligns with the mechanical loading pattern observed in our case.

Owing to the anatomical proximity of the hamstring origin to the sciatic nerve, PHT is frequently misdiagnosed as piriformis syndrome or sciatica [1]. Lempainen et al. also reported that deep gluteal and posterior thigh pain in PHT often mimics piriformis syndrome or lumbar radiculopathy, contributing to delayed or incorrect diagnosis in clinical practice [2]. Imaging findings may also be minimal, and MRI signal changes at the tendon insertion can be subtle; therefore, dynamic ultrasonographic evaluation is particularly useful [4]. Ultrasonographic findings in proximal hamstring tendinopathy can be subtle, and a mildly hypoechoic area without definite tendon thickening or tearing has been described in previous studies. Zissen et al. reported that early-stage tendinopathy may present only with focal or mild hypoechogenicity, with tendon thickening not always evident. Therefore, the ultrasonographic appearance in our case is consistent with published features for early or mild PHT [5].

PHT is considered a multifactorial condition, with repetitive tensile and compressive loading on the tendon regarded as the primary mechanism [1]. In this case, anterior pelvic tilt and limited hip extension associated with high-heel walking, combined with prolonged sitting during desk work, likely increased mechanical stress on the tendon. Anterior pelvic tilt has been reported to increase the contact pressure between the ischial tuberosity and hamstring tendon, thereby enhancing compressive stress at the enthesis [6].

Sustained hip flexion increases the tensile load on the tendon and contributes to the development of tendinopathy. In particular, direct compression of the ischial tuberosity against the seat surface during sitting can impose additional pressure on the tendon insertion and provoke symptom recurrence [2].

Ultrasound-guided local injection has been reported to be a safe and effective treatment for PHT, including injections of local anesthetics, corticosteroids, and platelet-rich plasma, with accurate needle placement at the ischial tuberosity being essential for pain relief [4]. In this case, immediate pain resolution was achieved, and symptoms remained stable when combined with conservative therapy.

However, because multiple interventions were implemented concurrently, including corticosteroid and local anesthetic injections, stretching exercises, hip-lift strengthening, activity modification, and reduction in prolonged sitting, it is not possible to determine the individual contribution of each component. The patient’s improvement likely resulted from the combined effects of this multimodal management approach rather than from the injection alone.

In addition, chronic buttock pain has a broad differential diagnosis beyond piriformis syndrome and lumbar pathology. Clinicians should also consider conditions such as ichiofemoral impingement, deep gluteal syndrome, sacroiliac joint dysfunction, gluteus medius or minimus tendinopathy, and referred pain from hip joint disorders. Awareness of these entities is important for improving diagnostic accuracy in patients presenting with persistent buttock pain.

Because the injection was performed in close proximity to the sciatic nerve, potential secondary effects should be considered, including transient irritation or compression due to injectate spread or increased pressure in fibrotic tissue. However, our patient underwent 18 ultrasound-guided injections without any neurological symptoms or other complications, suggesting that the procedure can be performed safely when carefully monitored.

Conservative management typically includes stretching, strengthening, and postural correction, with an emphasis on tendon-specific loading at increased muscle length and lumbopelvic stabilization exercises [7]. In this case, hip-lift exercises and stretching helped prevent recurrence. Although emerging treatments such as platelet-rich plasma and extracorporeal shockwave therapy have been investigated, the current evidence remains limited [7]. Corticosteroid injections are considered effective for short-term pain relief, consistent with the immediate improvement observed in this case.

This case represents a rare report of PHT in a nonathlete successfully treated with a local injection. PHT can develop not only in athletes but also in individuals exposed to chronic tendon loading during daily or occupational activities. Sustained anterior pelvic tilt, prolonged hip flexion, and extended sitting can impose compressive and tensile stress on the tendon insertion and should be recognized as potential etiological factors.

In pain clinics, awareness of PHT remains limited; however, it should be considered in the differential diagnosis of buttock pain. When differentiation from piriformis syndrome or lumbar pathology is difficult, characteristic findings, such as ischial tuberosity tenderness and symptom provocation during sitting, are particularly informative. Ultrasound-guided injection also has diagnostic value, as immediate pain relief following injection supports the diagnosis. Although an MRI of the ischial tuberosity was not obtained, the combination of localized tenderness, positive provocation tests, and characteristic ultrasonographic findings, even if subtle, was sufficient to support the diagnosis of PHT in accordance with previous reports. However, because a dedicated pelvic or proximal hamstring MRI was not performed, the diagnostic certainty remains limited, and in this case, the diagnosis should be regarded as presumptive rather than definitively confirmed by imaging.

Further studies on PHT in nonathletes are needed to establish optimal diagnostic and therapeutic strategies.

## 4. Conclusions

This case highlights that proximal hamstring tendinopathy can occur even in nonathletes when daily activities impose repetitive tensile and compressive loading on the tendon. Characteristic clinical findings such as ischial tuberosity tenderness, pain provocation during sitting, and positive provocation tests are essential for differentiating PHT from piriformis syndrome or lumbar pathology, particularly when imaging findings are subtle. Ultrasound-guided injection at the ischial tuberosity provided both diagnostic value and effective symptom relief in this patient, especially when combined with stretching, strengthening, and activity modification. Clinicians should consider PHT in patients presenting with buttock pain associated with lifestyle-related mechanical stress.

## Data Availability

The data supporting this case report are not publicly available due to patient privacy but are available from the corresponding author on reasonable request.

## Abbreviations

The following abbreviations are used in this manuscript:

PHT	Proximal hamstring tendinopathy
MRI	Magnetic resonance imaging
NRS	Numerical rating scale
